# C-reactive Protein and Biliary Complications as Independent Predictors of Hospital Stay in Acute Cholecystitis

**DOI:** 10.7759/cureus.87598

**Published:** 2025-07-09

**Authors:** Admir Abdic, Minela Becirovic, Emir Becirovic, Fuad Pasic, Zlatan Mehmedovic, Semir Hadžić, Mirha Agic, Amir Bećirović, Mirza Babic, Nadina Ljuca, Zarina Babic Jusic, Kenana Ljuca

**Affiliations:** 1 Department of Surgery, Cantonal Hospital Bihać, Bihać, BIH; 2 Internal Medicine Clinic, Department of Nephrology, University Clinical Centre Tuzla, Tuzla, BIH; 3 Internal Medicine Clinic, Intensive Care Unit, University Clinical Centre Tuzla, Tuzla, BIH; 4 Department of General and Abdominal Surgery, University Clinical Centre Tuzla, Tuzla, BIH; 5 Department of Endocrinology, University Clinical Centre Tuzla, Tuzla, BIH; 6 Department of Internal Medicine, Cantonal Hospital Bihać, Bihać, BIH; 7 Faculty of Medicine, University of Tuzla, Tuzla, BIH; 8 Department of Radiology, Cantonal Hospital Bihać, Bihać, BIH; 9 Department of Gynecology and Obstetrics, University Clinical Centre Ljubljana, Ljubljana, SVN

**Keywords:** acute cholecystitis, choledocholithiasis, c-reactive protein, inflammatory markers, length of stay, mirizzi syndrome

## Abstract

Background

Acute cholecystitis (AC) is a frequent surgical emergency associated with significant variability in clinical outcomes and hospital length of stay (LOS). Early identification of patients at risk for prolonged hospitalization can improve triage and resource planning. Inflammatory markers such as C-reactive protein (CRP), white blood cell count (WBC), and total bilirubin (TBil), along with biliary complications like choledocholithiasis and Mirizzi syndrome, may have prognostic value.

Materials and methods

This retrospective study included 150 patients who underwent cholecystectomy for AC at the Department of General and Abdominal Surgery, University Clinical Centre Tuzla, Tuzla, Bosnia and Herzegovina, between January 1, 2024, and January 31, 2025. Demographic, laboratory, and intraoperative data were collected. Receiver operating characteristic (ROC) analysis identified optimal cut-offs for inflammatory markers predicting prolonged LOS (≥7 days). Multivariate linear regression was used to assess independent predictors, including CRP, WBC, TBil, and intraoperative findings.

Results

We found that CRP was significantly higher in patients with prolonged LOS and demonstrated the highest predictive accuracy, with an area under the curve (AUC) of 0.733 (95% CI: 0.630-0.835), followed by TBil and WBC. In multivariate analysis, only CRP ≥110.5 mg/L (p<0.001), the presence of choledocholithiasis in 26 patients (17.3%; p=0.010), and Mirizzi syndrome in seven patients (4.7%; p=0.017) remained significant predictors. WBC and TBil lost significance after adjustment.

Conclusion

CRP is the most reliable independent laboratory predictor of prolonged LOS in AC. The presence of choledocholithiasis and Mirizzi syndrome further contributes to extended hospitalization. These factors should be considered in early clinical risk assessment.

## Introduction

Gallstone disease affects an estimated 10-20% of adults worldwide and remains a major source of surgical morbidity [1]. Its most frequent acute presentation, acute cholecystitis (AC), results from gallstone impaction in the cystic duct, leading to gallbladder distension, inflammation, and secondary bacterial infection. Untreated, AC may progress to serious complications like gangrene, perforation, or sepsis, all considered life-threatening conditions. Patients typically present with right-upper-quadrant pain, fever, nausea or vomiting, and a positive Murphy's sign [2]. Laboratory findings often reveal leukocytosis of various degrees and elevated inflammatory markers, while mild hyperbilirubinemia may signal concomitant biliary obstruction [3]. AC continues to be one of the leading indications for emergency abdominal surgery, and it is more prevalent in females and the geriatric population [4].

The Tokyo Guidelines 2018 (TG18) standardize AC diagnosis and stratify severity into mild, moderate, and severe categories based on clinical, laboratory, and imaging criteria, thereby guiding management, from early laparoscopic cholecystectomy to percutaneous drainage or delayed surgery. Regardless of severity, initial medical treatment, including antibiotics and fluid resuscitation, should be started immediately [5]. Although the grading system has proven clinical utility, TG18 staging does not invariably predict postoperative recovery [6].

Elevated values of widely available and inexpensive inflammatory biomarkers, including C-reactive protein (CRP), white blood cell count (WBC), and total bilirubin (TBil), have been linked to severe disease, postoperative complications, and prolonged hospital stay. Incorporating these parameters may refine risk stratification beyond TG18 grading [7].

Hospital length of stay (LOS) is a core outcome reflecting both clinical recovery and resource utilization. Prolonged LOS keeps patients in the hospital beyond the expected recovery period, inflating healthcare costs, heightening exposure to hospital-acquired infections, and occupying beds and staff that would otherwise serve new surgical cases [8]. Identifying predictors of extended hospital stay is therefore important from both clinical and organizational perspectives. Among factors prolonging LOS, common bile duct (CBD) stones and Mirizzi syndrome (MS) warrant particular attention [9]. CBD stones often necessitate endoscopic retrograde cholangiopancreatography or surgical exploration. MS is a rare complication of cholelithiasis seen in 0.63-5.7% of cholecystectomies [10]. In this condition, a stone gets impacted in the gallbladder neck or cystic duct, which extrinsically compresses or erodes into the common hepatic bile duct or CBD, producing obstructive jaundice, frequently forcing conversion to open surgery [11].

This retrospective cohort study evaluated whether admission CRP, WBC, TBil, CBD stones, MS, and operative findings could predict prolonged hospital stay after cholecystectomy for AC, aiming to refine early risk stratification and guide perioperative management.

## Materials and methods

We conducted a single-center, retrospective observational cohort study in the Department of General and Abdominal Surgery, University Clinical Centre Tuzla, Tuzla, Bosnia and Herzegovina. All consecutive adult patients who underwent cholecystectomy for AC between January 1, 2024, and January 31, 2025, were screened. The diagnosis of AC was confirmed according to the TG18 criteria [5]. Of 162 cholecystectomies performed for suspected AC during the study window, 12 cases were excluded (eight patients had chronic cholecystitis, three patients had gallbladder malignancy, and one patient had incomplete medical records), yielding a final cohort of 150 patients. An additional five patients who underwent intraoperative conversion from laparoscopic to open surgery were excluded from comparative analysis due to heterogeneous intraoperative indications and inability to classify them within a single operative category. A formal sample size calculation was not undertaken; all eligible cases were included to maximize statistical power.

Data was extracted from the electronic medical records and included demographics, comorbidities, TG18 severity grade, operative details, and LOS. Admission laboratory parameters were analyzed in the central laboratory by standardized assays and included CRP, WBC, TBil, urea, creatinine, glucose, hemoglobin, hematocrit, and erythrocyte count.

Two attending surgeons assigned a TG18 grade on admission; discrepancies were resolved by consensus. Operative findings were documented immediately postoperatively by the attending surgical team and included the presence of CBD stones, MS, case urgency (emergency vs. elective), surgical approach (laparoscopic, primary open, or conversion), and pathologic features that included gangrenous, perforated, fistulous, and hydrops gallbladder, empyema, peritonitis, and abscess formation.

The primary outcome was LOS. Patients were divided into two groups for comparative analysis: those with a hospital stay shorter than seven days and those with a stay of seven days or more, which corresponded to the median LOS in our cohort. In our institutional experience, patients with uncomplicated cholecystitis are typically discharged within 3-5 days. Therefore, a seven-day threshold also reflects the upper limit of expected recovery time and allows us to identify patients with prolonged hospitalization.

The study was approved by the Ethics Committee of the University Clinical Centre Tuzla (approval number: 02-09/2-208-3/24). Given its retrospective design and use of anonymized data, informed consent was waived. All extracted data were de-identified and stored on a password-protected institutional server in compliance with local data protection regulations.

Statistical analysis

Statistical analyses were conducted using jamovi (Version 2.6, The jamovi project, 2023, https://www.jamovi.org). The distribution of continuous variables was assessed using the Shapiro-Wilk test. As all continuous variables deviated from a normal distribution, non-parametric methods were applied. Continuous variables are presented as medians with interquartile ranges (IQR), while categorical variables are expressed as frequencies and percentages.

Group comparisons for continuous outcomes, including time to surgery and LOS, were performed using the Brunner-Munzel test, which is appropriate for data with unequal variances. Associations between categorical variables, such as TG18 grade, surgical approach, and comorbidity profiles concerning LOS, were assessed using Pearson's chi-squared test.

Spearman's rank correlation was used to evaluate relationships between admission laboratory parameters and LOS. To assess the predictive performance of CRP, WBC, and TBil for prolonged LOS (defined as seven days or longer), receiver operating characteristic (ROC) curve analysis was performed. The area under the curve (AUC) was calculated along with 95% confidence intervals, and optimal cut-off values were determined using the Youden index [12].

A multiple linear regression model was constructed to identify independent predictors of LOS. Dichotomized predictors were entered into the model based on clinically relevant thresholds, along with the presence of choledocholithiasis and MS. Prior to model fitting, assumptions of normality, linearity, homoscedasticity, and multicollinearity were evaluated. As violations of normality and homoscedasticity were detected, robust standard errors and bias-corrected and accelerated (BCa) bootstrap confidence intervals were applied to ensure reliable coefficient estimates. Statistical significance was defined as a p-value of <0.05.

## Results

The study cohort comprised 150 patients with a median age of 60 years (IQR 50-70.8), ranging from 21 to 89 years; 82 (54.7%) were female. According to TG18 criteria, 38 patients (25.3%) had Grade I, 95 (63.3%) Grade II, and 17 (11.3%) Grade III AC. The most common comorbidities were hypertension (96, 64%), obesity (33, 22%), hyperlipidemia (44, 29.3%), chronic heart disease (29, 19.3%), chronic kidney disease (14, 9.3%), and bronchial asthma (8, 5.3%) (Table 1).

**Table 1:** Baseline demographic and clinical characteristics of patients with acute cholecystitis TG18: Tokyo Guidelines 2018; IQR: interquartile range

Variable	Total (n=150)
Age, years, median (IQR)	60 (50-70.8)
Age range, years	21-89
Sex, n (%)	Male: 68 (45.3%); female: 82 (54.7%)
TG18 severity grade, n (%)	Grade I: 38 (25.3%); Grade II: 95 (63.3%); Grade III: 17 (11.3%)
Hypertension, n (%)	96 (64%)
Hyperlipidemia, n (%)	44 (29.3%)
Obesity, n (%)	33 (22%)
Cardiovascular disease, n (%)	29 (19.3%)
Chronic kidney disease, n (%)	14 (9.3%)
Bronchial asthma, n (%)	8 (5.3%)

Right-upper-quadrant pain was reported by nearly all patients across all TG18 grades, while nausea was present in 131 (87.3%) and vomiting in 120 (80%) in total. Fever was uncommon. No statistically significant differences were observed in symptom distribution across TG18 grades (Table 2).

**Table 2:** Distribution of symptoms on admission by TSG TSG: Tokyo Severity Grade

Symptom	TSG Grade I (n=38; 25.3%)	TSG Grade II (n=95; 63.3%)	TSG Grade III (n=17; 11.3%)	χ²	P-value
Pain	38 (100%)	94 (98.9%)	17 (100%)	0.583	>0.05
Nausea	30 (78.9%)	86 (90.5%)	15 (88.2%)	3.30	>0.05
Vomiting	26 (68.4%)	81 (85.3%)	13 (76.5%)	4.96	>0.05
Fever	1 (2.6%)	14 (14.7%)	3 (17.6%)	4.35	>0.05

**Figure 1:**
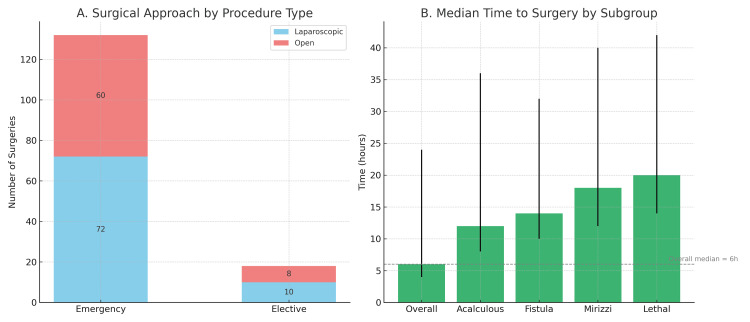
(A) Distribution of laparoscopic and open cholecystectomy in emergency and elective procedures. Open surgery was significantly more common in emergency cases with Grade II/III disease (p<0.001). (B) Median time from admission to surgery in the overall cohort and selected high-risk subgroups. Patients with acalculous cholecystitis, fistula, Mirizzi syndrome, and fatal outcomes experienced significantly longer delays (all p<0.05)

Emergency surgery was performed in 132 patients (88%), while 18 patients (12%) underwent elective cholecystectomy. Among those who underwent elective surgery, 13 patients (72.2%) had TG18 Grade I, four (22.2%) had Grade II, and one (5.6%) had Grade III disease. In contrast, the emergency group included 25 patients (18.9%) with Grade I, 91 (68.9%) with Grade II, and 16 (12.1%) with Grade III severity. Among emergency cases, 72 patients (48%) underwent laparoscopic cholecystectomy, while 60 (40%) required open laparotomy. Elective procedures included 10 laparoscopic (6.6%) and eight open surgeries (5.3%). The choice of surgical approach in elective cases did not differ significantly across TG18 grades (p=0.358), whereas in emergency surgeries, open procedures were significantly more frequent in patients with TG18 Grade II and III disease (p<0.001) (Figure 1A).

The median time from admission to surgery was six hours (IQR 4-24). In elective cases, surgery was performed during the same hospital stay following clinical stabilization. No patients were discharged and re-admitted for delayed cholecystectomy. Patients with acalculous cholecystitis, fistulous cholecystitis, MS, or fatal outcomes experienced significantly longer delays to surgery (all p<0.05) (Figure 1B).

All intraoperative findings were significantly associated with prolonged hospitalization (LOS ≥7 days), with the strongest associations observed for gangrenous cholecystitis, perforation, peritonitis, and abscess formation (all p<0.001), while other complications such as fistulous cholecystitis, MS, empyema, hydrops, and choledocholithiasis were also linked to extended LOS (p=0.003-0.018). Overall, severe or complicated intraoperative findings correlate with a significantly increased likelihood of prolonged hospitalization (Table 3).

**Table 3:** Intraoperative findings associated with prolonged hospitalization LOS: length of stay

Intraoperative finding	N (%) with LOS ≥7 days	Association with LOS	P-value
Gangrenous cholecystitis	27 (90%)	↑ longer LOS	<0.001
Perforated cholecystitis	10 (83.3%)	↑ longer LOS	<0.001
Fistulous cholecystitis	6 (85.7%)	↑ longer LOS	0.010
Hydrops	11 (73.3%)	↑ longer LOS	0.018
Empyema	13 (81.2%)	↑ longer LOS	0.005
Peritonitis	19 (90.5%)	↑ longer LOS	<0.001
Choledocholithiasis	20 (76.9%)	↑ longer LOS	<0.001
Mirizzi syndrome	6 (85.7%)	↑ longer LOS	0.003
Abscess formation	8 (88.9%)	↑ longer LOS	<0.001

**Figure 2:**
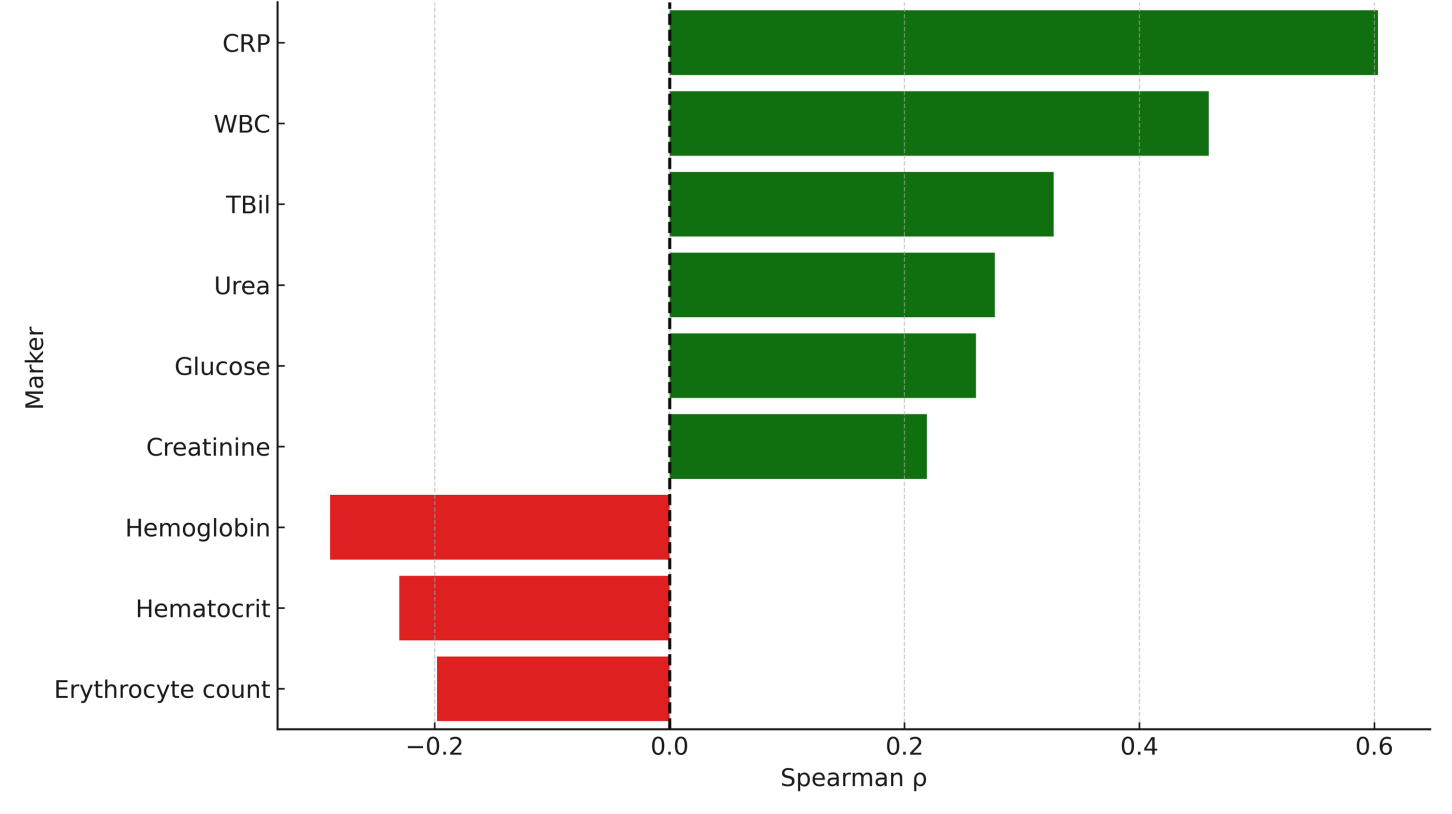
Spearman's correlation between laboratory markers and hospital stay CRP showed the strongest positive correlation with LOS (ρ=0.603; p<0.01), followed by WBC (ρ=0.459; p<0.01) and TBil (ρ=0.327; p<0.001). Urea (ρ=0.277; p<0.001), glucose (ρ=0.261; p=0.001), and creatinine (ρ=0.219; p<0.01) also showed significant positive correlations. In contrast, hemoglobin (ρ=-0.289; p<0.001), hematocrit (ρ=-0.230; p<0.01), and erythrocyte count (ρ=-0.198; p<0.05) were negatively correlated with LOS. CRP: C-reactive protein; WBC: white blood cell count; TBil: total bilirubin; LOS: length of stay

Spearman's rank correlation analysis revealed significant positive associations between LOS and several admission laboratory markers. CRP had the strongest correlation (ρ=0.603; p<0.01), followed by WBC (ρ=0.459; p<0.01) and TBil (ρ=0.327; p<0.001). Weaker but still significant correlations were noted for urea (ρ=0.277; p<0.001), glucose (ρ=0.261; p=0.001), and creatinine (ρ=0.219; p<0.01). In contrast, hemoglobin (ρ=-0.289; p<0.001), hematocrit (ρ=-0.230; p<0.01), and erythrocyte count (ρ=-0.198; p<0.05) were inversely correlated with LOS (Figure 2).

**Figure 3:**
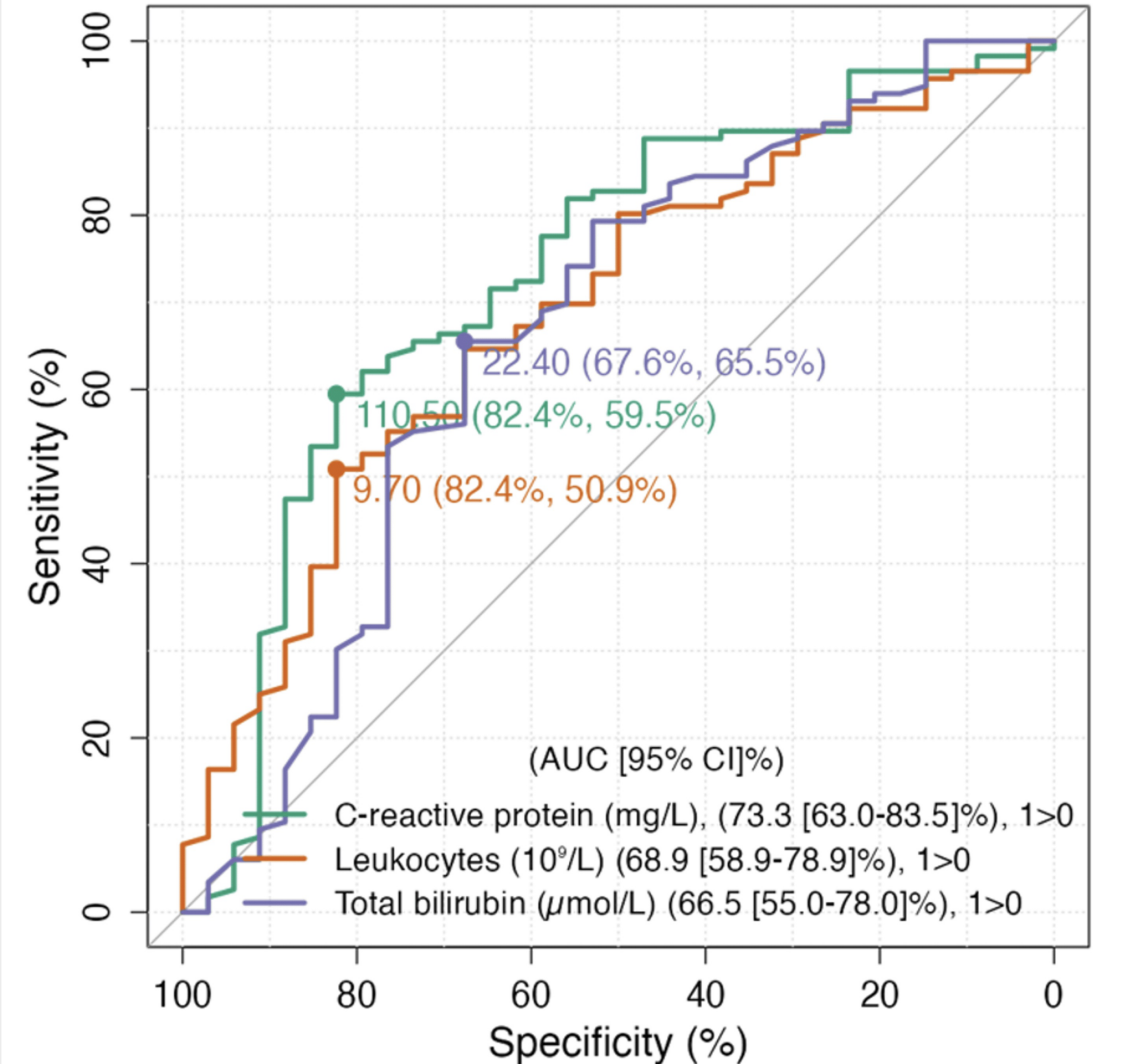
ROC curve analysis of admission CRP, leukocyte count, and total bilirubin for predicting prolonged hospitalization (≥7 days). A CRP level ≥110.5 mg/L showed the highest discriminative performance with an AUC of 0.733 (95% CI: 0.630-0.835), a sensitivity of 82.4%, and a specificity of 59.5%. Leukocyte count ≥9.7×10⁹/L had an AUC of 0.689 (95% CI: 0.589-0.789), while total bilirubin ≥22.4 µmol/L had the lowest predictive accuracy with an AUC of 0.665 (95% CI: 0.550-0.780) ROC: receiver operating characteristic; CRP: C-reactive protein; AUC: area under the curve

No significant correlations were found for temperature, blood pressure, heart rate, liver enzymes, gamma-glutamyl transferase (GGT), amylase, alkaline phosphatase, or platelet count. ROC analysis was performed to evaluate the diagnostic performance of CRP, WBC, and TBil in predicting prolonged LOS (Figure 3).

CRP ≥110.5 mg/L yielded the highest AUC of 0.733 (95% CI: 0.630-0.835), with a sensitivity of 82.4% and a specificity of 59.5%. WBC ≥9.7×10⁹/L had an AUC of 0.689 (95% CI: 0.589-0.789), while TBil ≥22.4 µmol/L showed an AUC of 0.665 (95% CI: 0.550-0.780). A multiple linear regression model identified independent predictors of LOS. The model was statistically significant (F(4, 141)=68.40; p<0.001), explaining 66% of the variance (adjusted R²=0.650). CRP ≥110.5 mg/L (B=2.35; 95% CI: 1.42-3.21; p<0.001), choledocholithiasis (B=15.31; 95% CI: 4.34-19.12; p=0.010), and MS (B=11.46; 95% CI: 5.00-21.49; p=0.017) were identified as significant predictors of prolonged LOS. WBC ≥9.7×10⁹/L did not retain significance in the multivariate model (p=0.139) (Table 4).

**Table 4:** Multiple linear regression model for predictors of hospital stay duration CRP: C-reactive protein; CI: confidence interval; SE: standard error; df: degrees of freedom Note: Inferential tests and p-values are adjusted for heteroscedasticity using robust standard errors. The intercept represents the baseline expected hospital stay in patients without the listed risk factors.

Predictor	Estimate (B)	SE	95% CI lower	95% CI upper	β	df	t	P-value
Intercept	5.500	0.235	5.089	5.869	0.000	141	23.375	<0.001
CRP ≥110.5 mg/L	2.345	0.472	1.421	3.213	0.271	141	4.973	<0.001
Leukocyte ≥9.7×10⁹/L	0.683	0.460	-0.175	1.464	0.078	141	1.487	0.139
Choledocholithiasis	15.309	5.875	4.340	19.115	0.411	141	2.606	0.010
Mirizzi syndrome	11.463	4.740	5.000	21.490	0.432	141	2.419	0.017

These findings underscore the prognostic relevance of CRP and biliary complications in predicting extended hospitalization following a cholecystectomy for AC.

## Discussion

While several intraoperative findings were significantly associated with prolonged hospitalization, CRP ≥110.5 mg/L emerged as the most reliable independent laboratory predictor of prolonged hospitalization in patients undergoing cholecystectomy for AC, increasing LOS by a mean of 2.4 days. WBC and TBil did not retain significance after adjustment.

This finding suggests that CRP more accurately reflects the extent of systemic inflammation than other routinely measured markers. CRP rises proportionally to IL-6-mediated hepatocyte stimulation and mirrors both systemic and local biliary inflammation more directly than leukocytosis or cholestatic indices [13]. Prior studies support these observations. A 2021 retrospective study including 176 patients with AC found that CRP values >100 mg/L were independently associated with gangrenous inflammation and extended LOS [14]. Another study of 804 emergency cholecystectomies demonstrated that CRP was a better predictor of operative difficulty than WBC, which lost significance in multivariate models [15]. Our study extends these findings by confirming the superior predictive value of CRP for LOS using ROC analysis, where it achieved the highest AUC among evaluated markers.

Although WBC is a component of the TG18 grading system, its predictive performance for LOS was notably limited in our cohort, possibly due to the influence of medications, comorbidities, or stress-related fluctuations. While TBil tends to rise in patients with CBD stones or MS, its overall prognostic value remained modest. Interestingly, recent data from cholangitis populations suggest that combined markers such as the neutrophil-to-lymphocyte ratio (NLR) may offer greater predictive insight than bilirubin alone [16]. A retrospective study of patients with AC or biliary colic found that TBil was significantly associated with bile duct stones, but bilirubin trends over time did not enhance predictive accuracy, unlike CRP, which may offer stronger clinical utility [17].

Our data also highlight the substantial impact of biliary complications. The presence of choledocholithiasis and MS was significantly associated with prolonged LOS. A multicenter retrospective analysis showed that patients with choledocholithiasis often required additional interventions such as endoscopic retrograde cholangiopancreatography, particularly in two-stage management protocols, leading to longer hospitalization [18]. Similarly, MS was associated with delayed recovery. One cohort study reported a conversion rate to open surgery of 64.6% in Mirizzi type I, reflecting increased operative complexity, prolonged duration, and greater postoperative care needs [19]. Open surgical procedures were significantly more frequent in TG18 Grade II and III cases, highlighting how disease severity influences operative approach and may contribute to prolonged recovery. Intraoperative findings such as gangrenous or perforated gallbladder were significantly associated with extended LOS in univariate analysis, but lost significance when adjusted for CRP and biliary complications [20]. This suggests that preoperative CRP and imaging can anticipate much of the intraoperative severity [21]. A 2023 study demonstrated that patients with gangrenous cholecystitis had mean CRP levels exceeding 230 mg/L, underscoring its prognostic utility [22].

These results have practical implications. Measuring CRP on admission offers a low-cost, widely accessible tool for early risk stratification [23]. In particular, a CRP level ≥30 mg/L demonstrated 97% sensitivity and 76% specificity for predicting complicated AC, supporting prioritization of early surgery, specialist input, and close monitoring [24]. Incorporating CRP into risk models may help clinicians better identify patients at risk for complicated disease and conversion to open surgery [25]. These insights can support more efficient surgical scheduling, optimize bed management, and improve communication with patients regarding expected recovery trajectories.

Limitations

This study has several limitations. The retrospective, single-center design introduces the risk of selection bias and unmeasured confounding, including individual surgeon preferences. We did not evaluate post-discharge readmissions, which may have led to an underestimation of the total burden of disease. The fixed LOS threshold of seven days may not fully reflect the spectrum of clinical complexity. Intraoperative findings were not stratified in full detail, and postoperative complications were not analyzed as separate outcome predictors. Although multivariate adjustment was applied, the possibility of residual confounding cannot be excluded. Additionally, differential leukocyte counts were not consistently available for all patients, precluding analysis of derived indices such as the NLR, which may hold independent prognostic value.

While TG18 severity grades were recorded, we did not perform a formal comparison between TG18 stage and inflammatory markers such as CRP, which may limit the assessment of their added prognostic value. Furthermore, we did not conduct subgroup analyses comparing elective and emergency patients in terms of LOS or biomarker levels, given the small number of elective cases and limited statistical power. Surgical approach was determined by the attending surgeon based on intraoperative judgment, anatomical complexity, and anticipated technical feasibility. As such, we were unable to systematically identify the clinical factors that influenced the choice between laparoscopic and open surgery within individual TG18 grades. In addition, five patients who required intraoperative conversion from laparoscopic to open surgery were excluded from subgroup analysis, which may have limited the representation of technically challenging cases. A larger, prospective multicenter study is needed to confirm and expand upon these findings.

## Conclusions

In patients undergoing cholecystectomy for AC, CRP was the most consistent and accurate predictor of prolonged hospitalization. Elevated CRP at admission reflected a higher inflammatory burden and was associated with more complex intraoperative pathology and increased postoperative care. Biliary complications, including CBD stones and MS, also contributed significantly to LOS. In contrast, WBC and TBil demonstrated weaker predictive capacity after adjustment. These results support the incorporation of CRP into early preoperative risk assessment. Combined with clinical and imaging data, CRP can aid in optimizing triage, surgical planning, and hospital resource utilization.
